# Association of Mps one binder kinase activator 1 (MOB1) expression with poor disease‐free survival in individuals with non‐small cell lung cancer

**DOI:** 10.1111/1759-7714.13608

**Published:** 2020-08-25

**Authors:** Nobuhisa Ando, Kentaro Tanaka, Kohei Otsubo, Gouji Toyokawa, Yuki Ikematsu, Maako Ide, Yasuto Yoneshima, Eiji Iwama, Hiroyuki Inoue, Kayo Ijichi, Tetsuzo Tagawa, Yoichi Nakanishi, Isamu Okamoto

**Affiliations:** ^1^ Research Institute for Diseases of the Chest, Graduate School of Medical Sciences Kyushu University Fukuoka Japan; ^2^ Department of Surgery and Science Graduate School of Medical Sciences, Kyushu University Fukuoka Japan; ^3^ Pathophysiological and Experimental Pathology Graduate School of Medical Sciences, Kyushu University Fukuoka Japan; ^4^ Kitakyushu City Hospital Organization Fukuoka Japan

**Keywords:** Hippo pathway, immunohistochemistry, lung adenocarcinoma, MOB1, vascular invasion

## Abstract

**Background:**

Mps one binder kinase activator 1 (MOB1) is a core component of the Hippo signaling pathway and has been implicated as a tumor suppressor. Here, we evaluated the possible relationship of MOB1 expression in non‐small cell lung cancer (NSCLC) to prognosis.

**Methods:**

We retrospectively analyzed 205 lung adenocarcinoma patients treated at Kyushu University Hospital between November 2007 and October 2012. MOB1 expression in tumor cells of surgical specimens was evaluated by immunohistochemistry. Invasive activity of NSCLC cell lines in vitro was measured with a transwell assay.

**Results:**

Expression of MOB1 was classified as high in 105 of the 205 (51.2%) tumor specimens, and such high expression was significantly associated with poor disease‐free survival (*P* = 0.0161). Among the various clinicopathologic parameters examined, high MOB1 expression was significantly associated only with intratumoral vascular invasion (*P* = 0.0005). Multivariate analysis also identified high MOB1 expression as a significant independent risk factor for disease‐free survival (*P* = 0.0319). The invasiveness of H1299 cells in vitro was increased or attenuated by overexpression or knockdown of MOB1, respectively.

**Conclusions:**

Our results suggest that MOB1 might promote early recurrence of NSCLC by increasing vascular invasion by tumor cells.

**Key points:**

**Significant findings of the study:**

We found that high MOB1 expression in surgical specimens of lung adenocarcinoma was associated with poor disease‐free survival and with intratumoral vascular invasion. MOB1 expression also promoted the invasiveness of NSCLC cells in vitro.

**What this study adds:**

Our results thus suggest that high MOB1 expression is a risk factor for early postoperative recurrence in lung adenocarcinoma.

## Graphical abstract

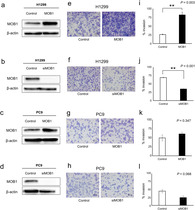

We found that high MOB1 expression in surgical specimens of lung adenocarcinoma was associated with poor disease‐free survival and with intratumoral vascular invasion. MOB1 expression also promoted the invasiveness of NSCLC cells in vitro. Our results suggest that MOB1 might contribute to early recurrence in NSCLC.

## Introduction

Despite the recent advances in targeted therapy for lung adenocarcinoma, non‐small cell lung cancer (NSCLC) remains the most common cause of cancer death worldwide.^1^ Individuals with early‐stage lung cancer who undergo curative surgical resection often experience recurrent disease or the development of distant metastases. Characterization of the signaling pathways associated with tumor progression would thus be expected to be of clinical value for the treatment of NSCLC.

The Hippo signaling pathway was initially identified in *Drosophila* and is highly conserved in mammals as a regulator of tissue homeostasis and regeneration, organ size, and tumorigenesis.^2^, ^3^ The Hippo pathway in mammals comprises four core components: mammalian Ste20‐like kinase (MST) 1/2, large tumor suppressor kinase (LATS) 1/2, salvador 1 (SAV1), and Mps one binder kinase activator 1 (MOB1). Activation of this core module results in the LATS1/2‐mediated phosphorylation and consequent cytoplasmic retention of the transcriptional coactivators YAP (Yes‐associated protein) and TAZ (transcriptional coactivator with PDZ binding motif). The nuclear exclusion of YAP‐TAZ blocks the TEAD (TEA domain‐containing) transcription factor‐mediated expression of various genes including those for growth factors and thereby results in tumor suppression.^4^, ^5^ Members of the nuclear Dbf2‐related (NDR) family of protein kinases in addition to LATS1/2 have also been recently identified as regulators of the core Hippo pathway.^6^

MOB1 is an adaptor protein that is highly conserved among eukaryotes and does not appear to contain any specific functional domain.^7^ Phosphorylation of MOB1 by MST1/2 promotes its binding to and consequent activation of LATS1/2 and thereby regulates YAP‐TAZ phosphorylation and subcellular localization.^8^, ^9^ MOB1 has been recognized as a tumor suppressor in various human cancer types including colorectal cancer,^10^ glioblastoma,^11^ and intrahepatic cholangiocarcinoma.^12^ The abundance of MOB1 mRNA has also been found to be reduced in NSCLC compared with normal lung tissue,^13^ but the clinical relevance of MOB1 expression in NSCLC has remained unclear.

We have now investigated the relation between clinicopathologic parameters and MOB1 expression in lung adenocarcinoma. Furthermore, we examined the effects of overexpression and depletion of MOB1 on the invasiveness of NSCLC cells. Our results suggest that expression of MOB1 might contribute to tumor promotion in lung cancer.

## Methods

### Patients and specimen collection

We retrospectively analyzed specimens from 205 individuals with lung adenocarcinoma who underwent surgical tumor resection between November 2007 and October 2012 at Kyushu University Hospital. Specimens were fixed in neutral‐buffered formaldehyde and processed for histopathologic and immunohistochemical evaluation. Histological subtype and pathological (p‐) stage were determined according to the WHO classification of 2004 and UICC guidelines for TNM classification, respectively. Histological diagnosis was confirmed by two pathologists. This study was approved by the ethics committee of Kyushu University.

### Immunohistochemical staining for MOB1

Paraffin‐embedded sections of surgically resected specimens were depleted of paraffin and rehydrated according to standard protocols before incubation overnight at 4°C with rabbit polyclonal antibodies to MOB1 (PA5‐14268; Thermo Fisher Scientific, Waltham, MA, USA) at a dilution of 1:200. Immune complexes were detected with biotin‐labeled secondary antibodies and streptavidin‐labeled horseradish peroxidase (Histofine SAB‐PO kit; Nichirei, Tokyo, Japan), with peroxidase activity being visualized with 3,3′‐diaminobenzidine. All immunohistochemical staining was evaluated separately by two investigators (N.A. and K.I.) without knowledge of the corresponding clinical records. Discrepant findings were reviewed and discussed until a consensus was obtained. Five fields were examined per slide, and 100 cells were evaluated per field at ×400 magnification. MOB1 expression was graded according to the percentage of tumor cells positive for cytoplasmic staining and the staining intensity as described previously^14^: 0 = complete absence of reactivity, 1 = weak cytoplasmic reactivity in ≤50% of cells, 2 = weak cytoplasmic reactivity in >50% of cells, and 3 = strong cytoplasmic reactivity. We classified 0 and 1 as low expression, and 2 and 3 as high expression.

### Cell culture

H1299 and PC9 cells were obtained from American Type Culture Collection (Manassas, VA, USA) and were cultured under a humidified atmosphere of 5% CO_2_ at 37°C in RPMI 1640 medium (Gibco, Carlsbad, CA, USA) supplemented with 10% fetal bovine serum (FBS).

### Immunoblot analysis

Cells were washed with ice‐cold phosphate‐buffered saline and then lysed by incubation at 95°C for five minutes in a solution containing 2% sodium dodecyl sulfate (SDS), 10% glycerol, 50 mM Tris‐HCl (pH 6.8), and a protease and phosphatase inhibitor cocktail (Nacalai tesque, Kyoto, Japan). The lysates were assayed for protein with the use of a DC Protein Assay Kit (Bio‐Rad, Hercules, CA, USA), and portions (30–50 μg of protein) were then fractionated by SDS‐polyacrylamide gel electrophoresis on a 10% (for E‐cadherin) or 15% (for other proteins) gel. The separated proteins were transferred to a polyvinylidene difluoride membrane, which was then incubated first overnight at 4°C with rabbit polyclonal primary antibodies and then for 50 minutes at room temperature with horseradish peroxidase–conjugated donkey secondary antibodies. (NA9340V; GE Lifesciences, Brøndby, Denmark). Primary antibodies included those to MOB1 (#3863, Cell Signaling Technology, Danvers, MA, USA) at a dilution of 1:1000, those to E‐cadherin (sc‐8426, Santa Cruz Biotechnology, Dallas, TX) at a dilution of 1:1000, those to Snail (C15D3; #3879, Cell Signaling Technology) at a dilution of 1:1000, and those to vimentin (#5741, Cell Signaling Technology) at a dilution of 1:1000. Antibodies to β‐actin (#4970S, Cell Signaling Technology) at a dilution of 1:1000 were used as a loading control. Immune complexes were detected with the use of Pierce Western Blotting Substrate Plus (Thermo Fisher Scientific) and a ChemiDoc XRS+ system (Bio‐Rad).

### Plasmid transfection

A full‐length human MOB1A cDNA (GenBank accession number NM_018221.5) was amplified from HEK293T by the polymerase chain reaction with the use of PrimeSTAR GXL DNA Polymerase (Takara Bio, Otsu, Japan). The amplification product was verified by sequencing and then ligated into the pcDNA3.1(−) vector (Thermo Fisher Scientific) between the Xho I and Kpn I sites with the use of an In‐Fusion HD Cloning Kit (Clontech, Mountain View, CA, USA). The resulting expression vector was introduced into H1299 or PC9 cells by transfection for 48 hours with the use of the Lipofectamine 3000 reagent (Thermo Fisher Scientific).

### RNA interference

Cells were plated at 60% to 70% confluence in six‐well plates and cultured for 24 hours before transient transfection for 48 hours with small interfering RNAs (siRNAs) mixed with the Lipofectamine RNAiMAX reagent (Thermo Fisher Scientific). The sequences of the siRNA duplexes (Japan Bio Services, Saitama, Japan) were 5′‐GACUAUUCUAAAGCGUCUGTT‐3′ and 5′‐CAGACGCUUUAGAAUAGUCTT‐3′ for MOB1A, 5′‐GUGCUCUGCACCAAAGUAUTT‐3′ and 5′‐AUACUUUGGUGCAGAGCACTT‐3′ for MOB1B, and 5′‐UUCUCCGAACGUGUCACGUTT‐3′ and 5′‐ACGUGACACGUUCGGAGAATT‐3′ for a control.

### Transwell invasion assay

Cell invasion was assessed with the use of a BioCoat Matrigel invasion chamber (#354480; Corning, Corning, NY, USA). Cells (5 × 10^4^) in 0.5 mL of serum‐free medium were added to the upper chamber of each well, and medium supplemented with 10% FBS as a chemoattractant was placed in the lower chamber. After culture for 22 hours, noninvading cells that remained on the upper surface of the membrane were removed by scraping, and cells that had migrated to the lower surface of the membrane were stained with Diff‐Quik solution (Sysmex, Kobe, Japan). The number of invading cells in five fields per membrane was then counted with a light microscope. The assay was performed in triplicate, and data are expressed as percent invasion through the Matrigel matrix and membrane relative to migration through a control membrane (#353097, Corning).

### Microarray analysis

The total RNA was isolated from H1299 cells using RNeasy Plus Mini Kit (Qiagen, Hilden, Germany) according to the manufacturer's instructions. RNA samples were quantified by an ND‐1000 spectrophotometer (NanoDrop Technologies, Wilmington, DE) and the quality was confirmed with a Experion System (Bio‐Rad Laboratories, Hercules, CA).

The cRNA was amplified, labeled, and hybridized to a 60K Agilent 60‐mer oligomicroarray according to the manufacturer's instructions. All hybridized microarray slides were scanned by an Agilent scanner. Relative hybridization intensities and background hybridization values were calculated using Agilent Feature Extraction Software (9.5.1.1).

Array name: SurePrint G3 Human Gene Expression Microarray 8x60K v3.

Labeling reagent: Agilent Low‐Input QuickAmp Labeling Kit, one‐color.

Raw signal intensities and Flags for each probe were calculated from hybridization intensities (gProcessedSignal), and spot information (gIsSaturated, etc), according to the procedures recommended by Agilent. (Flag criteria on GeneSpring Software. Absent (A): “Feature is not positive and significant” and “Feature is not above background”. Marginal (M): “Feature is not Uniform”, “Feature is Saturated”, and “Feature is a population outlier”. Present (P): others.)

The raw signal intensities of all samples were normalized by quantile algorithm with “preprocessCore” library package^15^ on Bioconductor software.^16^

We selected probes that call “P” flag at least one sample, excluding lincRNA probes. To identify up or downregulated genes, we calculated Z‐scores^17^ and ratios (nonlog scaled fold‐change) from the normalized signal intensities of each probe for comparison between control and experiment sample.

We then established criteria for regulated genes: (upregulated genes) Z‐score ≥ 2.0 and ratio ≥ 1.5‐fold, (downregulated genes) Z‐score ≤ −2.0 and ratio ≤ 0.66.

### Statistical analysis

The relationship between MOB1 immunoreactivity and clinicopathologic parameters was analyzed using a chi‐square test. Disease‐free survival (DFS) was measured from the date of surgery to the appearance of local or distant tumor progression. Overall survival (OS) was measured from the date of surgery to death or last follow‐up. DFS and OS were evaluated with the Kaplan‐Meier method, and the log‐rank test was applied to compare cumulative survival time between patient groups. The Cox proportional hazard model was used to determine univariate and multivariate hazard ratios for the study parameters. Parameters with a significant *P*‐value in univariate analysis were subjected to multivariate analysis. A *P*‐value of <0.05 was considered statistically significant. All statistical analysis was performed with JMP Pro version 14 software (SAS institute, Cary, NC, USA).

## Results

### MOB1 expression in lung adenocarcinoma specimens

**Figure 1:**
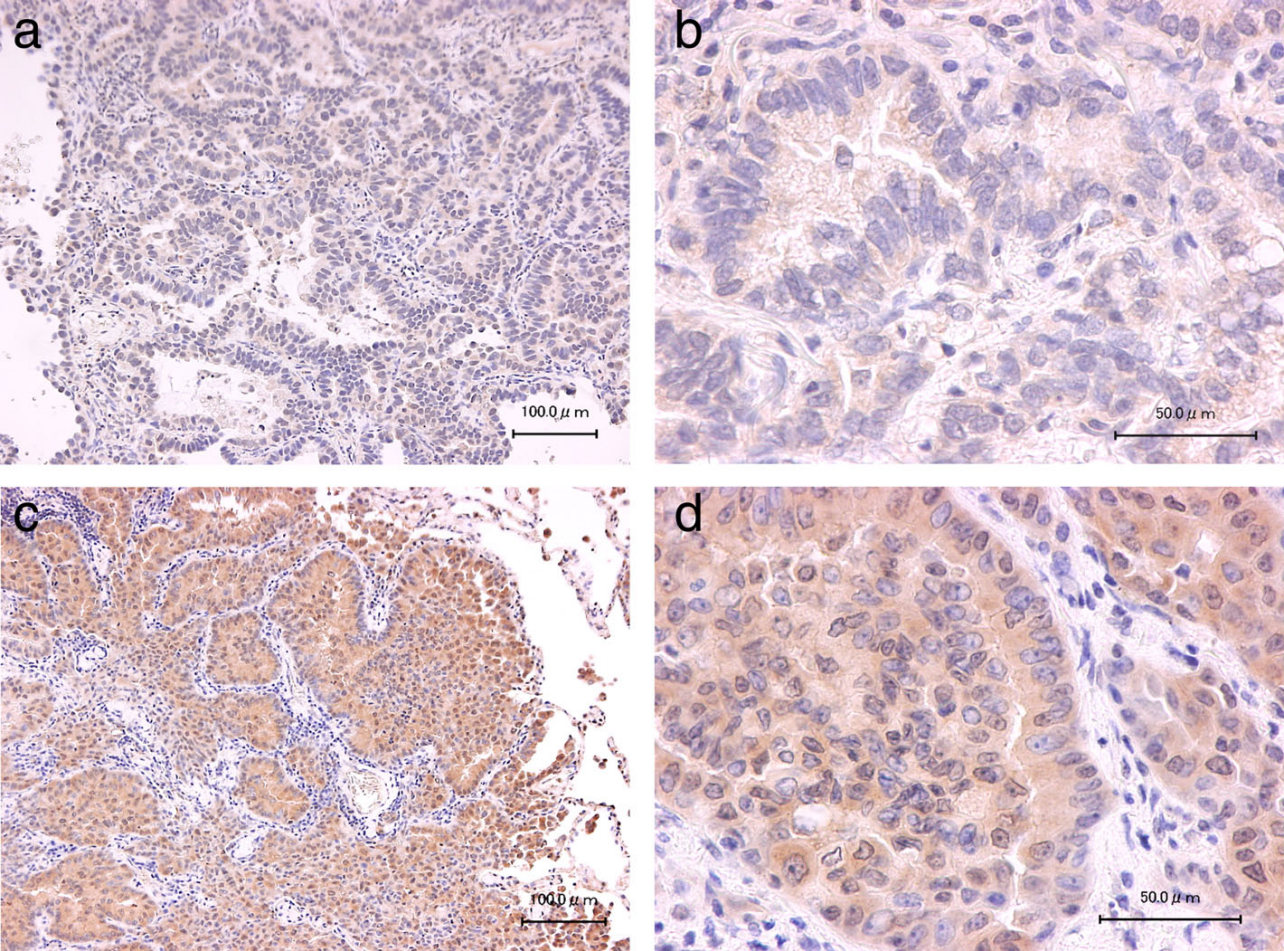
Representative immunohistochemical staining of MOB1 in lung adenocarcinoma specimens. Specimens negative (score = 0) (**a** and **b**) or positive (score = 4) (**c** and **d**) for MOB1 staining are shown at low (**a** and **c**) and high (**b** and **d**) magnification. Scale bars: 100 μm (**a** and **c**) or 50 μm (**b** and **d**).

We first examined the expression of MOB1 in 205 surgically resected lung adenocarcinoma specimens by immunohistochemistry (Fig 1). MOB1 expression was classified as high in 105 (51.2%) samples. The characteristics of the patients from whom the specimens were derived are shown in Table 1. These individuals included 100 males and 105 females, with an overall mean age ± SD of 67.2 ± 9.0 years, and 108 (52.7%) of them were former smokers. There were 159 (77.6%) cases of stage I (121 stage IA and 38 stage IB), 33 (16.1%) of stage II (18 stage IIA and 15 stage IIB), and 13 (6.3%) of stage III (11 stage IIIA and 2 stage IIIB) disease, with the predominance of early‐stage cases reflecting the fact that all specimens were obtained by surgical resection. A total of 91 (44.4%) patients were positive for *EGFR* mutation, and 12 (5.9%) for *EML4‐ALK* fusion.

**Table 1:** Patient characteristics (*n* = 205)

Characteristic	*n*
Age (years)	
<60	35
≥60	170
Sex	
Male	100
Female	105
Smoking history	
Former smoker	108
Never smoker	97
Pathological TNM stage	
IA	121
IB	38
IIA	18
IIB	15
IIIA	11
IIIB	2
T status	
T1	130
T2	60
T3	12
T4	3
N status	
N0	179
N1	14
N2	12
Gene mutation status	
None/unknown	102
*EGFR*	91
*EML4‐ALK*	12

### Relationship of MOB1 expression to DFS and vascular invasion

**Figure 2:**
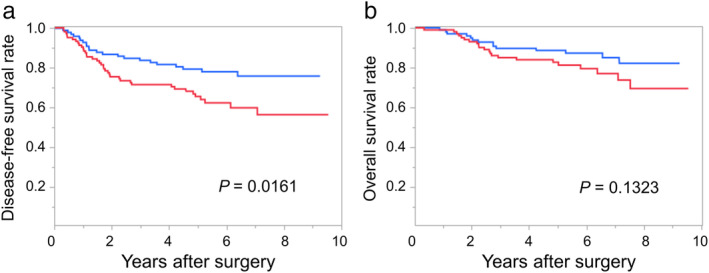
Kaplan‐Meier analysis of (**a**) disease‐free survival; and (**b**) overall survival for lung adenocarcinoma patients (*n* = 205) according to expression of MOB1 in tumor tissue (low or high) (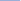 ) MOB1 low expression, (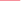 ) MOB1 high expression. The *P*‐values were determined by the log‐rank test.

To assess the relationship between MOB1 expression and prognosis of lung adenocarcinoma, we compared DFS and OS between low and high expression groups (Fig 2). DFS was significantly shorter in the MOB1‐high group than in the MOB1‐low group (*P* = 0.0161). OS also tended to be shorter in the MOB1‐high group than in the MOB1‐low group (*P* = 0.1323).

We next evaluated the relation between MOB1 expression and clinicopathologic parameters (Table 2). High expression of MOB1 was significantly related only to intratumoral vascular invasion (*P* = 0.0005) among the parameters examined.

**Table 2:** Relationships between MOB1 expression and clinicopathologic parameters

	MOB1 expression (*n*, %)				
Characteristic	Low	High	Total	*P*‐value	Oddsratio
Age (years)					
<60	20 (57.14)	15 (42.86)	35	0.3535	1.5000
≥60	80 (47.06)	90 (52.94)	170		
Sex					
Male	44 (44.00)	56 (56.00)	100	0.2092	1.4545
Female	56 (53.33)	49 (46.67)	105		
Smoking history					
Former smoker	52 (48.15)	56 (51.85)	108	0.8892	1.0549
Never smoker	48 (49.48)	49 (50.52)	97		
Pathological TNM stage					
Stage I	81 (50.94)	78 (49.06)	159	0.3151	1.4757
Stage II–III	19 (41.30)	27 (58.70)	46		
T status					
T0/T1	70 (53.85)	60 (46.15)	130	0.0608	1.7500
T2/T3/T4	30 (40.00)	45 (60.00)	75		
N status					
N0	90 (50.28)	89 (49.72)	179	0.2982	1.6180
N1/N2/N3	10 (38.46)	16 (61.54)	26		
Intratumoral lymphatic vessel invasion					
Negative	92 (51.11)	88 (48.89)	180	0.0888	2.2216
Positive	8 (32.00)	17 (68.00)	25		
Intratumoral vascular invasion					
Negative	84 (56.38)	65 (43.62)	149	0.0005	3.2308
Positive	16 (28.57)	40 (71.67)	56		

To investigate the association between clinicopathologic factors and survival, we performed univariate analysis (Table 3). MOB1 expression and p‐TNM stage were significantly associated with DFS, whereas only p‐TNM stage was associated with OS. An association of YAP expression with DFS or with OS was not observed. Multivariate analysis revealed that MOB1 expression (*P* = 0.0319) and p‐TNM stage (*P* < 0.0001) were significant independent risk factors for DFS, whereas only p‐TNM stage was such a factor for OS (Table 4).

**Table 3:** Univariate analysis of clinicopathological factors and survival

	Disease‐free survival			Overall survival		
Factor	HR	95% CI	*P*‐value	HR	95% CI	*P*‐value
MOB1 expression						
High vs. low	1.89	1.13–3.24	0.0157	1.66	0.86–3.33	0.1312
YAP1 expression						
High vs. low	0.77	0.45–1.39	0.3678	0.93	0.45–2.09	0.8415
Age (years)						
<60 vs. ≥60	1.39	0.70–3.18	0.3593	1.71	0.68–5.76	0.2762
Sex						
Male vs. female	1.33	0.80–2.22	0.2748	1.86	0.96–3.73	0.0654
Smoking history						
Former vs. never	1.05	0.63–1.75	0.8486	1.18	0.61–2.31	0.6201
Pathological TNM stage						
Stage I vs. stage II–III	4.81	2.88–8.04	<0.0001	3.66	1.89–7.08	0.0002

CI, confidence interval; HR, hazard ratio.

**Table 4:** Multivariate analysis of clinicopathological factors and survival

	Disease‐free survival			Overall survival		
Factor	HR	95% CI	*P*‐value	HR	95% CI	*P*‐value
MOB1 expression						
High vs. low	1.76	1.05–3.03	0.0319	1.52	0.78–3.05	0.2135
Pathological TNM stage						
Stage I vs. stage II–III	4.68	1.04–3.02	<0.0001	3.54	1.82–6.85	0.0003

CI, confidence interval; HR, hazard ratio.

Furthermore, we exploratorily analyzed the correlation of MOB1 expression with pathological subtypes in lung adenocarcinoma. In our study, most of the tissues were classified as papillary or lepidic‐dominant subtypes, while a small number of the acinar and solid types were observed. Therefore, we examined the correlation between papillary ‐ as well as lepidic‐dominant subtypes and MOB1 expression and revealed that there was no significant association of MOB1 expression with such subtypes. (Table S1).

### Effect of MOB1 expression on tumor cell invasiveness in vitro

**Figure 3:**
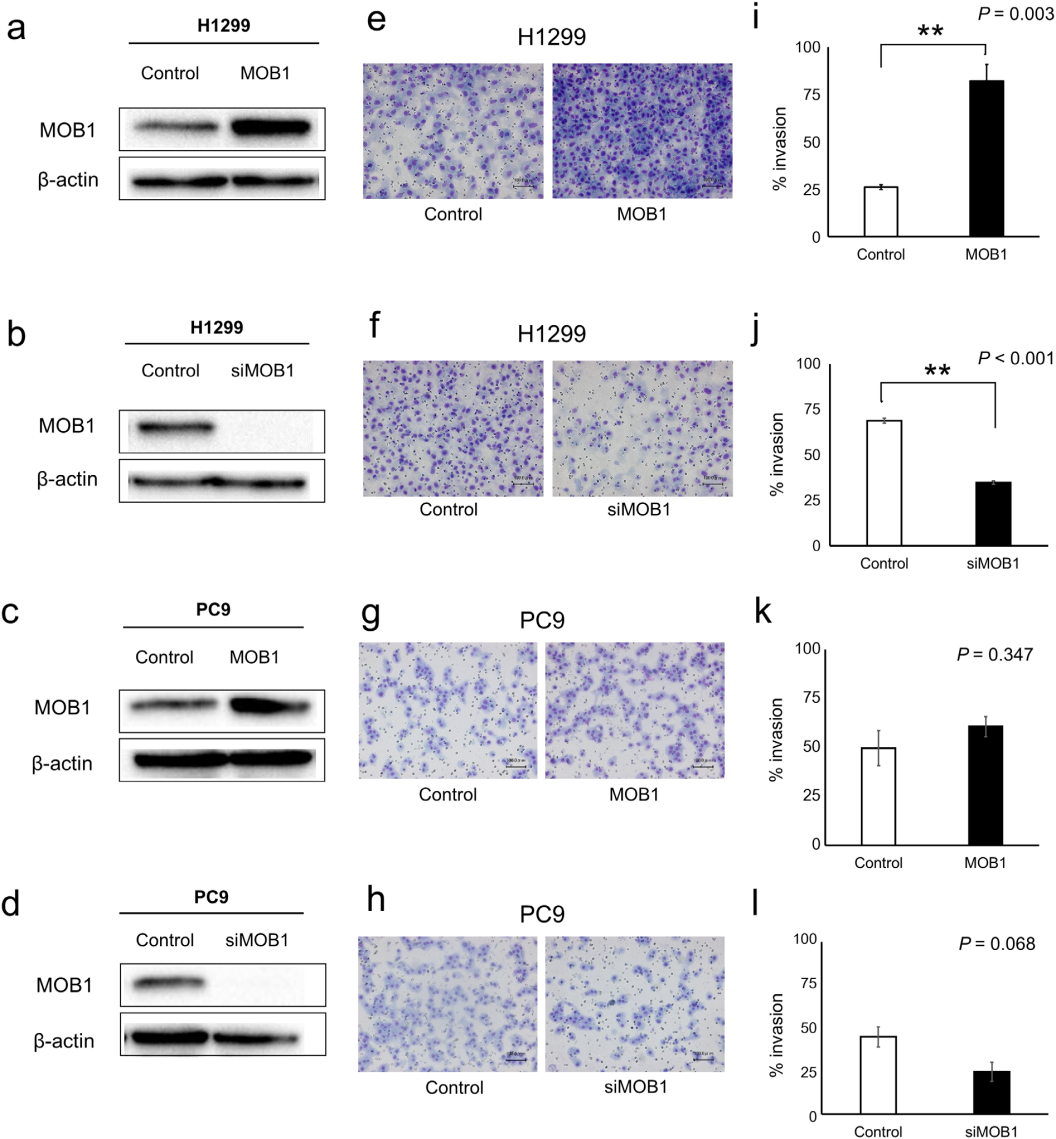
Effects of MOB1 expression level on tumor cell invasiveness in vitro. (**a**–**d**) Immunoblot analysis of MOB1 and β‐actin (loading control) in H1299 cells (**a** and **b**) or PC9 cells (**c** and **d**) that had been transfected either with an expression vector for MOB1A or the corresponding empty vector (control) (**a** and **c**) or with MOB1A/B or control siRNAs (**b** and **d**). (**e**–**h**) Cells as in (**a**) to (**d**), respectively, were assayed for invasion activity in vitro. Representative staining of invading cells on the lower side of the membrane is shown. Scale bars, 100 μm. (**i**–**l**) Percent invasion determined for cells as in (**e**) to (**h**), respectively. Data are means ± SD from three independent experiments. The *P*‐values were determined by Student's *t*‐test. **P < 0.01.

Vascular invasion is defined histologically as the presence of tumor cells within the lumen of blood vessels.^18^ Tumor cells must thus breach the basement membrane of vessels in order to enter the circulation. To determine whether MOB1 might contribute to vascular invasion by tumor cells, we performed a Matrigel invasion assay that is frequently adopted to examine the ability of cells to pass through a basement membrane.^19^, ^20^ The human NSCLC cell lines H1299 and PC9 were transfected either with an expression plasmid for MOB1 or with MOB1‐specific siRNAs. Efficient overexpression and knockdown of MOB1 were confirmed by immunoblot analysis (Fig 3a–d). Overexpression and knockdown of MOB1 resulted in a significant increase and decrease, respectively, in the invasion ability of H1299 cells (Fig 3e, f, i, and j). The invasiveness of PC9 cells was similarly affected by the level of MOB1 expression, although in this case the effects were not statistically significant (Fig 3g, h, k, and l). These results thus suggested that upregulation of MOB1 expression may contribute to the vascular invasiveness of NSCLC cells.

To elucidate how MOB1 expression could regulate invasion ability of lung cancer cells, we performed comprehensive gene analysis in MOB1‐overexpressd H1299 cells by microarray. The genes that were most significantly up‐ or downregulated (up to five genes) are listed in Table 5.

**Table 5:** List of genes whose expression levels are altered by forced expression of MOB1 in H1299 cells

Gene	Z‐score	Ratio
MOB1A	24.7973326	22.2779395
Invasion ‐ upregulation		
PGM5	3.43832363	2.32655678
MCAM	3.25742183	1.52536466
ADAM12	3.00796223	2.09257287
DCTN1	2.65505575	1.91837009
MCTS1	2.38808283	1.79628802
Invasion ‐ downregulation		
CUL4B	−13.043548	0.0122947
CCAR1	−7.1746088	0.52602177
ITGBL1	−3.6660045	0.40439721
MPP1	−2.7164825	0.51094315
TLR3	−2.6798701	0.51557135

Among the genes found to be greatly elevated, only a few studies have been conducted concerning PGM5 in cancer^21^, ^22^ and its function has remained undetermined. MCAM (also known as CD146, MUC18 or MelCAM) and ADAM12 have been known for its function in epithelial‐mesenchymal transition (EMT).^23^, ^24^, ^25^, ^26^ Since tumor invasiveness often correlates with EMT status, we hypothesized that MOB1 could be an EMT‐promoting factor. Then we investigated the correlation between MOB1 expression and three EMT‐related factors (E‐cadherin, Snail, and Vimentin) in H1299 cells that had been transfected with siMOB1 or control siRNAs. However, no significant differences were observed in the present results, suggesting that MOB1 expression dose not determine fate of EMT status in lung adenocarcinoma cells (Fig S1).

## Discussion

YAP and TAZ are transcriptional coactivators that are thought to promote the initiation or growth of most solid tumors.^27^ Other components of the Hippo pathway including MOB1 are thus implicated in suppression of tumor growth as a result of their inhibition of YAP‐TAZ activity. However, MOB1 is thought to contribute to various intracellular processes through formation of complexes with diverse binding partners such as serine‐threonine protein kinases, other enzymes, and scaffold proteins.^7^ Its role in cancer pathogenesis might therefore be expected to differ in a manner dependent on the expression of such partners in an organ‐specific manner.

We have now shown that high expression of MOB1 was significantly associated with poor DFS in individuals with lung adenocarcinoma, whereas an association of YAP expression with DFS or with OS was not observed. In addition, the expression of MOB1 in lung adenocarcinoma was significantly associated with only intratumoral vascular invasion among the clinicopathologic factors examined. Consistent with this association, we found that the level of MOB1 expression in NSCLC cells determined the invasiveness of these cells in vitro. Given that vascular invasion has been associated with disease recurrence in patients with NSCLC,^28^, ^29^ our results suggest that the positive regulation of vascular invasiveness by MOB1 in NSCLC cells might contribute to early recurrence.

A study based on mouse models recently showed that MOB1 contributes to the formation of lung tumors. Postnatal ablation of MOB1 thus attenuated the initiation of urethane‐induced lung adenoma.^30^ The interaction of MOB1 with other proteins may therefore play an indispensable role in the initiation and progression of NSCLC. MOB1 binds directly to other core components of the Hippo signaling pathway including MST1/2^31^ and LATS1/2^32^, ^33^ as well as to NDR1/2^34^ largely in a phosphorylation‐dependent manner. For example, phosphorylation of MOB1 on specific threonine residues by MST1/2 promotes its binding to LATS.^35^ Examination of the phosphorylation state of MOB1 may thus shed further light on its role in NSCLC.

According to recent reports, the expression of MOB1 rather inhibits cancer migration and invasion via phosphorylation of YAP. In hepatocellular carcinoma, MOB2 negatively regulates phosphorylation of MOB1 and LATS which could lead to YAP activation and increased motility of cancer cells.^36^ In another study of pancreatic ductal adenocarcinoma, lysine demethylase 2 (KDM2B) suppressed the expression of MOB1 by its binding to the promoter region of MOB1, then YAP‐mediated transcription promoted the invasion, migration and proliferation of cancer cells.^37^ Contrary to these reports, in our study MOB1 increased the migration ability of cancer cells. Our microarray results suggest that MOB1 in NSCLC could predominantly regulate the expression of genes promoting cancer invasiveness such as MCAM and ADAM12. It is possible that the expression of these genes might be induced by loss of the recently identified corepressor function of YAP‐TAZ.^38^ Indeed, we detected increased phosphorylation of YAP in MOB1‐overexpressing cells (unpublished data), indicative of YAP‐TAZ inactivation.

Alternatively, our results may reflect the operation of a YAP‐independent pathway for regulation of gene expression by MOB1. Phosphorylated MOB1 was thus found to bind to the protein DOCK1 in thymocytes^39^ and to regulate thymocyte egress and T cell survival^40^ in a YAP‐independent manner. In addition, in the regulation of neurite outgrowth, MOB1 degradation induced by the lipid phosphatase PTEN did not affect the phosphorylation of YAP or its nuclear translocation.^41^ Further studies will be required to investigate the molecular mechanism of action of MOB1 in lung cancer.

With regard to the limitations of our study, the tumor specimens analyzed reflected mostly early‐stage cancer, with limited histological characteristics. In addition, the clinical aspect of our study had a retrospective design and was based at a single institute, with the result that clinical and survival comparisons might have been influenced by selection bias.

We have shown that high expression of MOB1 in NSCLC was associated with early postoperative recurrence, possibly as a result of increased intratumoral vascular invasion by cancer cells. Further studies on the role of MOB1 as well as on that of other proteins of the Hippo signaling pathway in lung cancer may provide a basis for the development of new treatment strategies targeting this pathway.

## Disclosure

The authors declare no conflicts of interest associated with this manuscript.

## Supporting information


**Figure S1** Correlation between Mps one binder kinase activator 1 (MOB1) wxpression and EMT‐related dactors in H1299 xells. Immunoblot analysis of MOB1, E‐cadherin, Snail, Vimentin andβ‐actin (loading control) in H1299 cells that had been transfected with siMOB1 or control siRNAs.

**Table S1** Relationships between MOB1 expression and histological subtypes (*n* = 205).
