# Supplementary figures and images for: Association of Mps one binder kinase activator 1 (MOB1) expression with poor disease‐free survival in individuals with non‐small cell lung cancer
Source: Thorac Cancer. 2020 Aug 25;11(10):2830–9. doi: 10.1111/1759-7714.13608 (PMC7529568; doi:10.1111/1759-7714.13608)

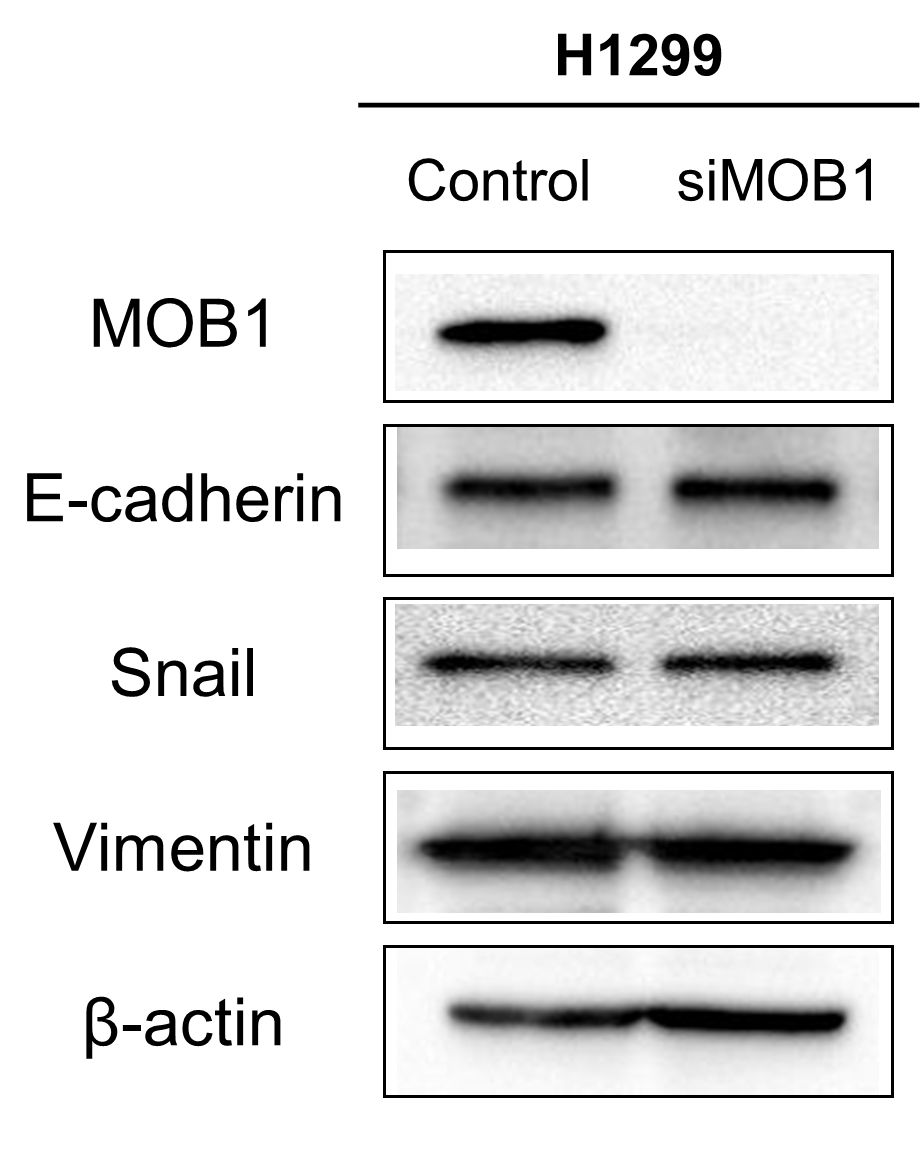

Supplement: Supplementary file 1 — Figure S1 Correlation between Mps one binder kinase activator 1 (MOB1) wxpression and EMT‐related dactors in H1299 xells. Immunoblot analysis of MOB1, E‐cadherin, Snail, Vimentin andβ‐actin (loading control) in H1299 cells that had been transfected with siMOB1 or control siRNAs. [file TCA-11-2830-s001.tif]
