# Supplementary material for: Association of Mps one binder kinase activator 1 (MOB1) expression with poor disease‐free survival in individuals with non‐small cell lung cancer
Source: Thorac Cancer. 2020 Aug 25;11(10):2830–9. doi: 10.1111/1759-7714.13608 (PMC7529568; doi:10.1111/1759-7714.13608)
Supplement: Supplementary file 2 — Table S1 Relationships between MOB1 expression and histological subtypes (n = 205). [file TCA-11-2830-s002.docx]

| **Table S1.** Relationships Between MOB1 Expression and Histological Subtypes (*n*=205) | | | | | |
| --- | --- | --- | --- | --- | --- |
| Characteristic | MOB1 expression (*n*, %) | | Total | *P*-value | Odds ratio |
|  | Low | High |  |  |  |
| Papillary predominant or other subtypes | | | | | |
| Papillary | 61 (44.53) | 76 (55.47) | 137 | 0.0568 | 1.6755 |
| Other | 39 (57.35) | 29 (42.65) | 68 |  |  |
| Lepidic predominant or other subtypes | | | | | |
| Lepidic | 31 (59.62) | 21 (40.38) | 52 | 0.9758 | 0.5565 |
| Other | 69 (45.10) | 84 (54.90) | 153 |  |  |
|  | | | | | |
